# Patients report inferior quality of care for knee osteoarthritis prior to assessment for knee replacement surgery – a cross-sectional study of 517 patients in Denmark

**DOI:** 10.1080/17453674.2019.1680180

**Published:** 2019-10-22

**Authors:** Lina H Ingelsrud, Ewa M Roos, Kirill Gromov, Sofie S Jensen, Anders Troelsen

**Affiliations:** a Department of Orthopedic Surgery, Copenhagen University Hospital Hvidovre, Hvidovre, Denmark;; b Department of Sports Science and Clinical Biomechanics, University of Southern Denmark, Odense, Denmark

## Abstract

Background and purpose — Clinical care pathways for knee osteoarthritis (OA) are not always in line with clinical guidelines. We investigated (1) the patient-perceived quality of OA management, (2) which physiotherapist-delivered treatments patients with knee OA have attempted, and (3) patients’ expected subsequent treatment, at the time of referral to an orthopedic surgeon.

Patients and methods — This cross-sectional study included all patients with scheduled first-time appointments for knee OA at an orthopedic outpatient clinic from April 2017 to February 2018. Postal questionnaires included the 16-item OsteoArthritis Quality Indicator (OA-QI) questionnaire and questions about physiotherapist-delivered treatment for knee OA.

Results — 517 of 627 (82%) eligible patients responded. Responders’ (63% female) mean age was 67 years. The mean pass rate for the 16 independent quality indicators was 32% (8–74%). Sub-grouped into 4 categories, pass rates for independent quality indicators ranged from 16–52% regarding information, 9–50% regarding pain and functional assessment, 8–35% regarding referrals, and 16–74% regarding pharmacological treatment. While half of responders felt informed of physical activity benefits, only one-third had consulted a physiotherapist during the past year. Commonest physiotherapist-delivered treatments were exercise therapy for 22% and participation in the Good Life with osteoArthritis in Denmark (GLA:D) program for12% of responding patients. 65% expected surgery as subsequent treatment.

Interpretation — Patients with knee OA are undertreated in primary care in Denmark; however, our findings may only reflect healthcare settings that are comparably organized. Our results call for better structure and uniform pathways for primary care knee OA treatment before referral to an orthopedic surgeon.

According to national and international clinical guidelines, first-line treatment strategies for knee osteoarthritis (OA) should have been offered prior to referral for knee replacement surgery (Danish Health Authority 2012, American Academy of Orthopaedic Surgeons 2013, Fernandes et al. 2013, National Clinical Guideline Centre 2014, Bannuru et al. 2019).

In Scandinavia, several implementation strategies have been undertaken to optimize the adherence to clinical guideline recommendations for non-operative treatment of OA (Thorstensson et al. 2015, Skou and Roos 2017, Moseng et al. 2019). In Denmark, the focus on patient education and exercise has been strengthened with the physiotherapist-led treatment program Good Life with Osteoarthritis in Denmark (GLA:D) that was launched in 2013. Results from the GLA:D registry are promising and show that patients experience pain relief, and improved physical function and quality of life after attending the program (Skou and Roos 2017). Clarifying what patients expect from their subsequent treatment may help understand what drives patients to seek referral to an orthopedic surgeon. We therefore (1) evaluated the patient-perceived quality of OA management, (2) described which physiotherapist-delivered treatments patients with knee OA have attempted, and (3) described the patients’ expectations of their subsequent treatment, at the point in time when patients are referred to an orthopedic surgeon in Denmark.

## Patients and methods

We followed the STROBE guidelines for the reporting of this study. In this cross-sectional study, we consecutively included patients from 1 outpatient orthopedic department at a public hospital in Denmark. The inclusion criterion was patients having been referred for first-time appointments with an orthopedic surgeon for an assessment of knee OA. Patients unable to speak or read Danish and patients who cancelled their consultation were excluded. In the period March 2018 to February 2019, patients were sent a postal invitation and were asked to respond to a self-reported questionnaire and return this in a pre-stamped envelope before the appointment with the orthopedic surgeon.

### Questionnaire

The questionnaire included the patient-reported OsteoArthritis Quality Indicator questionnaire (OA-QI). The 17-item OA-QI was developed in 2010 by Østerås et al. (2013, 2015), who found it to have acceptable validity and moderate reliability in patients with knee, hip, or hand OA. The OA-QI includes quality indicators related to patient education and information, assessment of pain and function, referrals, and pharmacologic treatment for OA. For this study, we adjusted the OA-QI to specifically reflect knee OA and removed the item regarding referral to an orthopedic surgeon.

We further asked about the number of physiotherapy consultations for knee OA the patient had attended during the past year. Patients who had consulted a physiotherapist were asked which types of treatments they had received from a predefined list and could add other treatments in free text, if relevant. Patients also responded to a question about their expectations of subsequent treatment following the consultation with the orthopedic surgeon. The following 6 response options were given: surgical intervention, exercise therapy recommendation, weight loss recommendation, pain intervention recommendation, no further treatment recommended, or other, with the opportunity to expand on other expectations in free text.

The patients’ knee pain and functional limitations due to knee problems were measured using the Oxford Knee Score (OKS). The OKS was developed in 1998 as an outcome measure for people having total knee replacement, and its reliability and validity characteristics were later confirmed in non-operatively treated patients with knee OA. The 12 items are each scored from 0 to 4, summed to a total score of 0 (worst) to 48 (best) (Dawson et al. 1998). Additionally, patients reported their average knee pain intensity during the past week on a 0–100 mm VAS scale ranging from no pain to worst pain imaginable. Finally, patient demographics—height, weight, age, education level, employment status, smoking, residential status, comorbidities, and symptom duration—were collected.

The degree of radiographic OA was evaluated for patients with routinely obtained anteroposterior, weight-bearing radiographs available, and classified with the Kellgren and Lawrence (KL) classification system, ranging from grade 0 to 4 OA. For patients who reported problems with both knees, the most severe KL grade was recorded.

### Pilot study

To test the feasibility of the questionnaire, we conducted a pre-test of the questionnaire on 6 patients who completed the questionnaire in the waiting room, prior to their consultation with an orthopedic surgeon. The patients were observed while completing the questionnaire, followed by a semi-structured interview to assess the feasibility, comprehensibility, and relevance of the questions. The time to complete the questionnaire ranged from 9 to 20 minutes. We subsequently conducted a pilot study to test the feasibility of the data collection procedure. Of 114 patients who were sent a postal questionnaire, only 52 (46%) responded and 3 items had more than 10% missing responses. Based on the pilot study, we made smaller wording edits to the items with large degrees of missing items.

### Statistics

Data are presented as mean (SD) for normally distributed continuous data and median with 25th and 75th quantiles for non-normally distributed data. Categorical data are presented as numbers and percentages. Results from the OA-QI are reported as pass rates for each of the 16 quality indicators separately, and as summary pass rates across all quality indicators for each patient, as described in the original publication. Separate quality indicator pass rates were calculated as the percentage of patients responding “yes” out of the total number responding either “yes” or “no,” combined, taking the relevance of the quality indicator into account. Summary pass rates for each individual patient were calculated as the percentage of indicators with the response “yes” out of the total number of indicators with responses “yes” or “no,” combined (Østerås et al. 2013). Statistical analyses were performed with the statistical package R, version 3.4.1 (R Foundation for Statistical Computing, Vienna, Austria).

### Ethics, funding, and potential conflicts of interest

The study was approved by the national data protection agency (Journal number AHH-2015-093). Since only questionnaire-based data were used, ethical committee approval was not required. The study was conducted in accordance with the WMA Declaration of Helsinki. The study was funded by the Danish Rheumatism Association and by the Orthopaedic Department at the Copenhagen University Hospital Hvidovre. ER is co-founder of GLA:D. The authors declare no other potential conflicts of interest.

## Results

**Figure 1.**
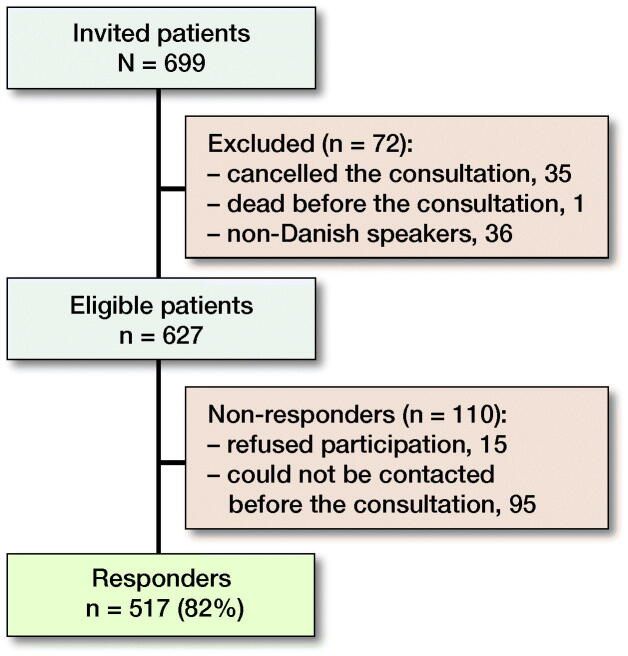
Flow diagram.

517 of 627 (82%) eligible patients responded (Figure 1). The responders had a mean (SD) age of 67 (11) and 63% were female. In comparison, the non-responders’ mean (SD) age was 63 (12) years and 65% were female (Table 1).

**Table 1.** Patient characteristics. Values are frequency (%) unless otherwise specified

Factor	Responders	Respons
Age, mean (SD)	515	67 (11)
Female	517	324 (63)
BMI, mean (SD)	503	30 (5)
Education level	498	
Primary		129 (26)
Secondary		141 (28)
Tertiary		228 (46)
Current smoker	515	74 (14)
Occupation	514	
Working full-time or part-time		141 (27)
Unemployed		28 (5)
Sick leave		23 (4)
Retired		322 (63)
Living alone	517	208 (40)
Comorbidities	517	
None		105 (20)
Heart disease		54 (10)
Hypertension		231 (45)
Cerebral vascular disease		18 (3)
Peripheral artery disease		62 (12)
Lung disease		55 (11)
Diabetes		58 (11)
Kidney disease		13 (3)
Neurologic disease		9 (2)
Liver disease		7 (1)
Cancer within 5 years		35 (7)
Depression		39 (8)
Spinal arthritis or other spinal condition		172 (33)
Other arthritides		92 (18)
Knee OA duration	512	
0–6 months		43 (8)
6–12 months		49 (10)
1–2 years		73 (14)
2–5 years		105 (21)
5–10 years		122 (24)
> 10 years		120 (23)
Knee pain (VAS) median (IQR)	486	7 (5–8)
OKS, mean (SD)	501	23 (8)
KL grade	459	
0		1 (0)
1		29 (6)
2		89 (19)
3		171 (37)
4		169 (37)

The number of missing items varied across the variables, thus the specific numbers of included observations are presented.

Abbreviations: BMI: body mass index, OA: osteoarthritis,

VAS: visual analogue scale, OKS: Oxford Knee Score,

KL grade: Kellgren and Lawrence classification system.

### Quality indicators for knee OA treatment

The mean pass rate for the 16 independent quality indicators was 32% (8–74) (Table 2). Sub-grouped into 4 categories, the pass rates were 16–52% related to OA information, 9–50% related to assessment of pain and function, 8–35% related to referrals, and 16–74% related to pharmacological treatment (Table 2). The median (25th to 75th quantile) summary pass rate, i.e., the percentage of fulfilled quality indicators overall per patient, was 29% (18–50). 1% of patients reported “yes” to all the quality indicators that were relevant to them, while 6% had not achieved any of the relevant quality indicators.

**Table 2.** Patient self-reported quality indicator pass rates for knee osteoarthritis treatment during the past year at the point of referral to an orthopedic surgeon for an evaluation of surgical appropriateness. Values are frequency (%) unless otherwise specified

OA-QI (n = 508)^a^	Yes	No		Missing	Pass rate (95% CI)^b^
	Do not remember				
1. Information about disease development	128 (25)	319 (63)	50 (10)	11 (2)	29 (25–33)
2. Information about treatment modalities	148 (29)	320 (63)	23 (5)	17 (3)	32 (28–36)
3. Information about self-management	72 (14)	390 (77)	28 (6)	18 (4)	16 (13–19)
4. Information about lifestyle adaptation	100 (20)	364 (72)	23 (5)	21 (4)	22 (18–26)
5. Information about physical activity	247 (49)	228 (45)	15 (3)	18 (4)	52 (48–56)
6. Referral for physical activity	167 (33)	310 (61)	12 (2)	19 (4)	35 (31–39)
	Not overweight				
7. Information about weight reduction	113 (22)	247 (49)	140 (28)	8 (2)	31 (27–36)
8. Referral for weight reduction	29 (6)	326 (64)	139 (27)	14 (3)	8 (6–11)
	Do not have this problem				
9. Assessment of problems in daily activities	117 (23)	319 (63)	48 (9)	24 (5)	27 (23–31)
10. Assessment for walking aid	72 (14)	335 (66)	84 (17)	17 (3)	18 (14–22)
11. Assessment for other daily living aids	33 (6)	345 (68)	109 (21)	21 (4)	9 (6–12)
	Do not have pain				
12. Assessment of pain	242 (48)	241 (47)	11 (2)	14 (3)	50 (46–55)
13. Recommended paracetamol	349 (69)	125 (25)	11 (2)	23 (5)	74 (69–77)
14. Offered stronger pain killers	182 (36)	276 (54)	23 (5)	27 (5)	40 (35–44)
15. Information about NSAIDS (side) effects	219 (43)	232 (46)	29 (6)	28 (6)	49 (44–53)
16. Offered joint injection	72 (14)	386 (76)	25 (5)	25 (5)	16 (13–19)
17. Referral to orthopedic surgeon	Not applicable in this study				

Abbreviations: OA-QI: OsteoArthritis Quality Indicator questionnaire.

a 9 of the 517 responders had not answered any of the questions in the OA-QI.

b Pass rates were calculated as the percentage of patients responding “yes” out of the total number responding either “yes” or “no.”

### Physiotherapist-delivered treatment

184 of 494 (37%) responders who completed the question regarding the number of physiotherapy consultations during the past year had had at least 1 consultation. Of these, 66 had seen the physiotherapist 1 to 3 times (Table 3).

**Table 3.** Number of physiotherapy consultations due to knee osteo­arthritis received during the past year prior to consulting with an orthopedic surgeon

Physiotherapy consultations^a^	n (%)
None	309 (63)
1–3	66 (13)
4–6	31 (6)
7–9	30 (6)
10–12	21 (4)
> 12	36 (7)

a 23 of 517 did not respond to this question (n = 494).

The 2 most frequently reported physiotherapist-delivered treatments were “any type of exercise therapy” for 22% and participation in the GLA:D program (the combination of patient education and supervised group-based neuromuscular exercise therapy) for 12% of the 513 patients who had completed the question. Furthermore, OA information, stretching, and massage where each reported by 9% of the responding patients (Table 4). Most patients had received several treatments in combination.

**Table 4.** Type of physiotherapist-delivered treatments for knee osteo­arthritis during the past year

		Percentage of^a^	
Physiotherapist-delivered treatment	n	total responders (n = 513)^b^	those consulting a physiotherapist (n = 184)
GLA:D participation	61	12	33
OA information	44	9	24
Any type of exercise	114	22	62
Stretching	45	9	24
Massage	47	9	26
Electrotherapy	24	5	13
Acupuncture	37	7	20
Insoles	34	7	18
Gait assessment	17	3	9
Other	43	8	23

Abbreviations: GLA:D: Good Life with Osteoarthritis in Denmark (the combination of patient education and supervised group-based exercise therapy).

a Percentages do not add up to 100% across the treatment types because some patients received several treatments in combination.

b 4 patients out of the total 517 responders did not answer the question about type of physiotherapist-delivered treatments.

### Patients’ expectations of treatment suggested by the orthopedic surgeon

Prior to their consultation with the orthopedic surgeon, the majority (65%) expected to be offered a surgical intervention, followed by expecting to be recommended exercise therapy (30%), pain management (22%), or weight loss intervention (15%) (Table 5).

**Table 5.** Patients’ expectations of their subsequent treatment after consulting with the orthopedic surgeon

Expectations^a^	n (%)^b^
Surgery	324 (65)
Exercise	150 (30)
Weight loss	76 (15)
Pain management	108 (22)
No treatment	22 (4)
Other	101 (20)

a 20 of 517 did not respond to this question (n = 497).

b Percentages do not add up to 100% because patients may have responded to several expectations.

## Discussion

We investigated the quality of care delivered in primary care for patients with knee OA, prior to referral for an orthopedic surgeon. We found that while quality indicators regarding pharmacological pain relief were fulfilled by one-third to two-thirds, most quality indicators relating to patient information, exercise, weight loss, and functional assessment were fulfilled for at most one-third of patients. Of special note is that less than 1 in 3 felt informed about the way the OA disease develops, possible treatment modalities, how to self-manage their disease, and how to change their lifestyle. Furthermore, even though 1 in 2 felt informed about the importance of physical activity and exercise, only 1 in 3 had consulted a physiotherapist during the past year before referral to an orthopedic surgeon.

Our results support prior studies reporting suboptimal quality of knee OA care in Denmark and other countries. In a study of hand, knee, and hip OA in a primary care setting in 1 municipality in Norway, the median summary pass rate was 27%, which is comparable to our finding of 29% (Østerås et al. 2013). Our results further confirm the results from the smaller (n = 49) Danish knee OA cohort in a study comparing the quality of knee OA care across 4 European countries (Østerås et al. 2015). In that study, quality indicators regarding OA information were fulfilled by only 17% to 38% in the Danish cohort, which is comparable to the 16% to 32% from our study. Furthermore, Østerås et al. found that around half the responders had received referrals for supervised exercise (i.e., physiotherapy) in Norway, Portugal, and the UK, while that proportion was only 21% in Denmark. In comparison, in our study, only 35% were referred, even though they were at a later stage of disease. These findings highlight that access to physiotherapists should be facilitated since physiotherapists have a core role in prescribing and supervising exercise therapy for patients with knee OA. A financial barrier may be present for patients in Denmark. Although patients can directly access private physiotherapy clinics they then have to pay the full treatment cost, while those with a referral from a general practitioner are reimbursed approximately 40%.

Skou et al. (2015b) suggested that knee replacement can be postponed, even in those with moderate to severe OA, and also that patients undergoing a structured and optimized non-operative treatment program may be better prepared for a surgical decision. In their clinical trial, comparing an optimized non-operative treatment strategy with an optimized non-operative treatment strategy combined with total knee replacement, only 26% of those in the non-operative group subsequently decided to undergo surgery during the following 12 months. Rheumatologists, orthopedic surgeons, and general practitioners considered that a barrier to referring patients with OA to physiotherapy is that patients receive non-evidence-based passive treatment modalities, instead of exercise therapy (Selten et al. 2017). Correspondingly, only 2 in 3 patients in our study had received exercise as part of their physiotherapy treatment. 10 years ago, Holden et al. (2009) found that physiotherapists in the UK were concerned about the real benefits of exercise for knee OA, and moreover many believed that exercise would potentially be harmful for the osteoarthritic joint. More recent studies have, however, established that patients with severe radiographic OA grades can also achieve pain relief and functional improvement with exercise (Juhl et al. 2014, Skou et al. 2015a), and even large exercise loads do not seem to be harmful for the cartilage in the osteoarthritic knee (Bricca et al. 2018). Even though a more recent report suggests that physiotherapists are convinced by this contemporary knowledge (Spitaels et al. 2016), another study pointed at several healthcare providers doubting the real benefits of exercise (Selten et al. 2017). Furthermore, lack of support, or conflicting information from different healthcare providers, were found to be important barriers for patients to adhere to exercise therapy (Kanavaki et al. 2017). Motivating patients to exercise is challenging if they believe that their joint is worn down, which may partly explain our finding of low adherence to exercise therapy. While providing patient education on OA and self-management strategies is crucial for long-term adherence, only 1 in 4 patients in our study had received information about OA from the physiotherapist. A lack of established self-management and patient education tools amongst physiotherapists (Holden et al. 2009) may explain why few patients experienced OA information as being part of the physiotherapist-delivered treatment. The GLA:D program does incorporate a structured approach and specific tools for patient education (Skou and Roos 2017); however, only 1 in 3 patients from our study who had consulted a physiotherapist had participated in the GLA:D program.

We found that 2 in 3 patients expected to be waitlisted for surgery at their consultation with the orthopedic surgeon. This may reflect that 1/3 of patients see the orthopedic surgeon as the OA expert providing a second opinion instead of a professional carrying out surgery. This proportion might have been higher had the OA education for general practitioners and physiotherapists been improved, and had the non-operative treatment strategy been optimized. This hypothesis is strengthened by our additional finding that 1 in 3 expected to be referred for exercise therapy. A commonly elaborated response to the question regarding expectations of the following consultation was that they hoped to “find out what is wrong with my knee” (data not shown). These statements may indicate that the patients had not received a clinical OA diagnosis in general practice and support the idea that the surgeon is seen as the OA expert.

Differences in healthcare systems across countries may limit the external validity of our findings. The generalizability is, however, strengthened by the high response rate of consecutively invited patients from a large-volume orthopedic department with a large area of uptake that includes both rural and urban settlement. Despite our consecutive approach, there is a risk of selection bias since non-responders were on average 4 years younger than the responders. A possible explanation could be that younger patients with knee problems often did not consider their OA diagnosis definitive, and therefore did not consider it relevant to answer the questionnaire. Further limitations involve a risk of recall bias (Basedow and Esterman 2015). Patients may have forgotten which specific type of physiotherapist-delivered treatment they had received, and the number of physiotherapy consultations. Furthermore, patients may have had previous 1st-time appointments due to their knee OA at other specialist centers than ours, or at our center due to OA in the opposite knee. However, we hypothesize that any previous specialist consultations would have led to an improved pass rate. Finally, there may be a discrepancy between the degrees of fulfilled quality indicators from the patients’ perspective, in comparison with the healthcare professional’s perspective. Quality assessment based on reviewing medical records is commonly used to reflect the professionals’ perspectives (Basedow and Esterman 2015). A shortcoming of this approach, however, is that it may lead to both under- or overestimating the usage of healthcare processes due to inaccurate information in the medical records (Luck et al. 2000).The self-reported OA-QI has demonstrated adequate content and construct validity (Østerås et al. 2013), and is a feasible option to capture patient-perceived quality of care.

In conclusion, patients with knee OA are undertreated in primary care; however, our findings may only reflect healthcare settings that are comparably organized. Only about 1/3 had consulted a physiotherapist during the last year, and only 1/4 were informed about the disease and its management options prior to seeing an orthopedic surgeon. Our results calls for better structure and uniform pathways for knee OA treatment in the primary sector before referral to an orthopedic surgeon. Future studies should investigate whether optimizing the quality of care in the primary health care sector has a positive effect on the outcomes of knee OA treatment across treatment sectors.
